# The American Dental Association

**Published:** 1870-06

**Authors:** W. H. Shadoan


### THE AMERICAN DENTAL ASSOCIATION.

Dr. Taft—*Dear Sir:* The Committee of Arrangements have made the following arrangements for the next meeting to be held in Nashville in August next. Dr. Morgan has secured the use of the Representative Hall for holding the meetings, and has secured a reduction of fare at the Maxwell House, one of the finest hotels on the continent, from the regular rates to from $2 50 to $3 50 per day—owing to the floor occupied. The Committee of Five appointed at the last annual meeting to secure half fare on the various railroads, have as yet made no report.

We do not know what they have done. They have been written to and requested to attend to the matter at once, and report, so that it may be published. I, as one of the Committee, have written to several General Ticket Agents on the subject, and have heard from four with the following result:

The Louisville and Nashville road cheerfully granted half fare. The Louisville and Cincinnati Short Line also agree to the arrangements. The Ohio and Mississippi Railroad declined after, as I so understood the General Ticket Agent at the Galt House in this city to assent to it. The Pan Handle route also declined, but any arrangements made with the Pennsylvania Central road will be complied with by the Pan-Handle route.

It is to be hoped that Dr. Buckingham, at Philadelphia, and Dr. Bogue, at New York, will see to it that the Pennsylvania Central come into the arrangements. I have also written to the proprietors of the Mammoth Cave, which lays near the Louisville and Nashville Railroad, in reference to arrangements for our accommodation there; also transportation from the railroad to the cave.

We hope to be able to make favorable arrangements with all railroads North and South for half fare. The roads will charge full fare going and return free, when commutation arrangements are made.

The Committee are determined to make this one of the most pleasant meetings ever held, and are constantly at work with that view. We hope that all the old members will attend, and as many new ones as are appointed delegates.

As soon as other additional facts are known, you will be apprised of them. All Dental Journals are requested to publish this letter, and anything else of interest on the subject.

W. H. Shadoan, one of the Committee.

